# Surveillance and Clinical Characterization of Influenza in a University Cohort in Singapore

**DOI:** 10.1371/journal.pone.0119485

**Published:** 2015-03-19

**Authors:** Aidan Lyanzhiang Tan, Ramandeep Kaur Virk, Paul Anantharajah Tambyah, Masafumi Inoue, Elizabeth Ai-Sim Lim, Ka-Wei Chan, C. Senthamarai Chelvi, Say-Tat Ooi, Catherine Chua, Boon-Huan Tan

**Affiliations:** 1 Department of Medicine, National University of Singapore, Singapore, Singapore; 2 Agency for Science, Technology and Research, Singapore, Singapore; 3 Detection & Diagnostics Laboratory, DSO National Laboratories, Singapore, Singapore; 4 Department of General Medicine, Khoo Teck Puat Hospital, Singapore, Singapore; 5 University Health Centre, National University of Singapore, Singapore, Singapore; 6 Department of Epidemiology and Public Health, National University of Singapore, Singapore, Singapore; The University of Hong Kong, HONG KONG

## Abstract

**Background:**

Southeast Asia is a potential locus for the emergence of novel influenza strains. However, information on influenza within the region is limited.

**Objectives:**

This study was to determine the proportion of influenza-like illness (ILI) caused by influenza A and B viruses in a university cohort in Singapore, identify important distinctive clinical features of influenza infection and potential factors associated with influenza infection compared with other causes of ILI.

**Methodology:**

A surveillance study was conducted from 2007 to 2009, at the University Health and Wellness Centre, National University of Singapore (NUS). Basic demographic information and nasopharyngeal swabs were collected from consenting students and staff with ILI, with Influenza A and B identified by both culture and molecular methods.

**Results:**

Proportions of influenza A and B virus infections in subjects with ILI were 153/500 (30.6%) and 11/500 (2.2%) respectively. The predominant subtype was A/H1N1, including both the seasonal strain (20/153) and the pandemic strain (72/153). The clinical symptom of fever was more common in subjects with laboratory confirmed influenza than other ILIs. On-campus hostel residence and being a student (compared with staff) were associated with increased risk of laboratory confirmed influenza A/H1N1 2009 infection.

**Conclusions:**

This study provides a baseline prevalence of influenza infection within young adults in Singapore in a university setting. Potential risk factors, such as hostel residence, were identified, allowing for more targeted infection control measures in the event of a future influenza pandemic.

## Introduction

The influenza virus is a major cause of morbidity and mortality worldwide. In Singapore, it causes significant economic impact, estimated to include more than 3 million doctor visits and 2 million lost days of work [1]. Singapore does not have a well-defined influenza seasonality, and influenza infection tends to occur throughout the year. [2–3]

Many respiratory pathogens can present with “influenza-like” symptoms. Infections caused by other respiratory pathogens may present similarly to influenza infections, making it difficult to distinguish them clinically [4]. Hence, accurate detection and subtyping of influenza virus are important for epidemiologic surveillance [5], while aiding infection control and management for the individual and public health responses to influenza outbreaks and pandemics [6–7].

Transmission of influenza-like illnesses (ILIs) has been historically known to occur more easily in relatively closed populations such as campus accommodations, dormitories or military camps. Recently, a study in Singapore defined the proportion of influenza-like illnesses in a military setting due to actual laboratory confirmed influenza infection in 2010 [8]. However, there has not been a similar study done in civilian populations in recent years. University students may have an advantage for surveillance as local students reflect local community epidemiology while overseas students studying in Singapore may introduce new strains from their home country. This was evidenced in 1968, where good characterization of the 1968 influenza pandemic was obtained from students and staff of the National University of Singapore attending the University Health Centre (UHC) [9]. The potential for a student health centre acting as sentinel surveillance site has not been thoroughly explored since then in Singapore or other Southeast Asian cities to our knowledge.

It has been postulated that those living within hostels, being a relatively closed community with close contact, have a higher risk of influenza transmission. Similarly, individuals clustered in academic centers or schools within the campus that are located close together in physical location may be at higher risk of influenza transmission from their fellow staff or students.

We conducted a prospective surveillance study in a university cohort to determine the proportion of ILI actually caused by influenza A and B viruses [10] and to better understand the risk factors for laboratory confirmed influenza virus infection in this cohort.

## Materials and Methods

The methods for this study have previously been described in a descriptive analysis and molecular characterization of viruses circulating on campus [10]. The study details are as below.

### Study Population

Students and staff from the National University of Singapore (NUS) seek medical attention at the UHC. Individuals meeting the case definition for ILI of fever with respiratory symptoms [11] were invited to participate in the study.

### Ethics Statement

The study was approved by the NUS Institutional Review Board (IRB). The NUS-IRB reference number is 06–156 and approval number is NUS-282. Written informed consent was obtained from all the participants before sample and data collection. Students were generally aged between 17 to 25 and considered under NUS-IRB to be able to give consent without parental participation.

### Sample and Data Collection

Individuals had two nasopharyngeal swabs collected by a trained research assistant. Viruses were subsequently isolated using two different culturing methods and two different molecular methods including inoculation in embryonated chicken eggs, followed by Madin-Darby Canine Kidney (MDCK, American Type Culture Collection ATCC, CCL-34, Rockville, MD, USA) cells; immunofluorescence assay for influenza A virus antigens and multiplex End-point RT-PCR and pyrosequencing to obtain influenza subtypes. Basic demographic and clinical information were also collected.

### Statistical Analysis

Data were analysed with STATA 12th Edition, to obtain confidence intervals and prevalence rate ratios. Sensitivity, specificity, positive predictive value (PPV) and negative predictive value (NPV) were calculated using standard formulas. Individuals with incomplete or invalid data of the variable being analyzed were excluded from the respective analyses. Bonferroni correction was used to account for multiple comparisons.

## Results

A total of 500 subjects' data were analyzed. Characteristics of the study population are summarized in table 1. Gender was approximately equal, 48% female (240/500) and 52% male (260/500). The age of the subjects ranged from 17 years to 70 years with a median of 22 years. Overall, 30.6% (153/500) tested positive for the presence of influenza A virus and 2.2% (11/500) for influenza B virus as previously described [10]. The predominant subtype was influenza A/H1N1. The Singapore seasonal A/H1N1 made up 20 of the 92 cases (21.7%) of A/H1N1 with the remaining 72 being pandemic A/H1N1. 6 samples of Influenza were not typeable. The details are summarized in table 2.

**Table 1:** Characteristics of Study Population.

**Age (years)**	23	*Median*
*25% Percentile*	20	
*75% Percentile*	25	
**Gender**		
*Male*	260	52
*Female*	240	48
**Nationality**		
*Singaporean*	251	50.2
*Non-Singaporean*	249	49.8
**Smoking status**		
*Never smoked*	436	87.2
*Smoker / Ex-smoker*	64	12.8
**Occupation**		
*Student*	400	80.48
*Non-student*	97	19.52
Not stated *	3	
**Campus**		
*Life Science (Science/Nursing/Medicine)*	134	27.86
*Non-life science (Engineering/Computing/Finance)*	347	72.14
Not stated *	19	
**Domicile**		
*Hostel*	216	44.17
*Non hostel*	273	55.83
No valid address stated *	11	

Data is n (%)

* Not included within analysis

**Table 2:** Number (%) of Subjects positive for Influenza Virus Infection.

Influenza A	153	30.6
Seasonal H1N1	20	4
Pandemic H1N1	72	14.4
A / H3 N2	55	11
A / Not typeable	6	1.2
Influenza B	11	2.2

**Fig 1:**
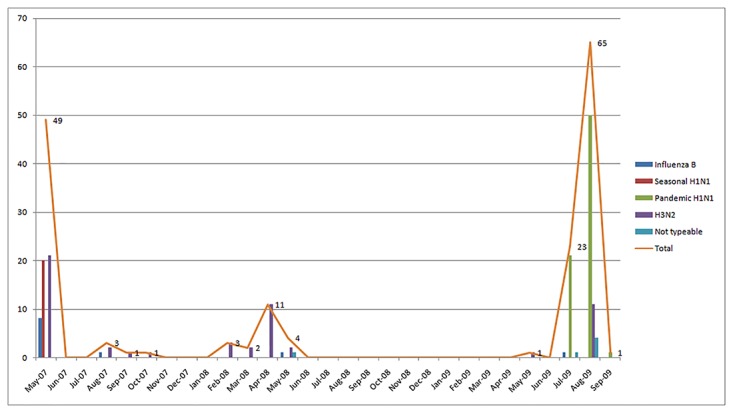
Influenza Distribution According to Time.

The distribution of Influenza was clustered across different time periods (Fig. 1) although there were gaps in the collection of data during the university vacations. Influenza B was isolated more often in early 2007. Subtype A/H3N2 was noted across the entire time period of sampling. The pandemic strain of A/H1N1 was isolated only in the later half of 2009 as would be expected.

Of the seven symptoms of ILI elicited, five were noted to have a significant association with laboratory confirmed influenza infection overall: muscle aches (odds ratio of 1.613), cough (1.425), stuffy or runny nose (1.327), chills (1.510) and fever (2.357). Only fever (3.255) was noted to be significantly associated in laboratory confirmed 2009 pandemic H1N1. The positive and negative predictive values of each symptom are summarized in table 3.

**Table 3:** Symptom distribution in subjects and prevalence rate ratios with predictive values.

Symptom	Number (%)	OR of Influenza	(95% CI)	PPV	NPV
Fever	280 (56.0)	**2.36**	(1.74–3.20)	0.43	0.81
Chills	214 (42.8)	**1.51**	(1.18–1.94)	0.4	0.73
Aches	246 (49.2)	**1.61**	(1.24–2.09)	0.4	0.74
Stuffy or runny nose	280 (56.0)	**1.33**	(1.02–1.73)	0.36	0.72
Sore throat	325 (65.0)	1.26	(0.96–1.67)	0.35	0.72
Cough	252 (50.4)	**1.43**	(1.10–1.84)	0.38	0.72
Hoarse voice	216 (43.2)	1.22	(0.95–1.57)	0.36	0.7

OR: Odds Ratio, PPV: Positive Predictive Value, NPV: Negative Predictive Value

Amongst the 500 subjects, 216 (44.17%) lived on campus in hostels at time of presentation and 273 did not. 11 did not provide a valid address or hostel location. Comparison between those living in hostels and not was not significant (*p* = 0.070) for overall influenza positivity disregarding type or subtype. However, on campus hostel residence was a significant risk factor for Influenza A infection (OR 1.31 [1.00–1.71], *p* = 0.043) and in particular for pandemic influenza A H1N1 2009 (OR 1.96 [1.25–3.08],*p* = 0.002).

Of the total of 500 subjects, 400 (80.4%) were students. 3 did not state their occupation. Students and staff with ILI had similar rates of laboratory confirmed influenza positivity (*p* = 0.662). However, students had a significantly larger proportion testing positive for pandemic Influenza A H1N1 2009 (OR 4.12 [1.54–11.0], *p* = 0.001).

Study subjects were also divided according to faculties they were involved with based on the geographical distribution of the different schools and faculties on campus; the life science part of campus namely, Medicine, Science and Nursing. Students from the non-life science part of campus were from Engineering, Business, Arts and Computing. A total of 481 subjects were able to be identified according to this categorisation. There was no association between the different parts of campus and the influenza types and subtypes detected.

Study subjects were classified according to their nationality into Singaporean (50.2%) and non-Singaporean (49.8%) groups. Nationality was used as a surrogate for travel history, given that the vast majority of overseas students at the university returned to their home countries during university vacations. Comparison between the two groups also showed no significant difference in influenza positivity. The results are summarized in table 4.

**Table 4:** Risk factors for Influenza Positivity.

Laboratory confirmed Positivity of:	Influenza	Influenza A	Influenza A / Seasonal H1N1	Influenza A / Pandemic H1N1	Influenza A / H3N2	Influenza A / Not typeable	Influenza B
	*Odds Ratio (95% CI)*	*Odds Ratio (95% CI)*	*Odds Ratio (95% CI)*	*Odds Ratio (95% CI)*	*Odds Ratio (95% CI)*	*Odds Ratio (95% CI)*	*Odds Ratio (95% CI)*
**Age**							
>25 years	0.900	0.841	1.103	**0.247**	**1.892**	0.662	1.892
	(0.658–1.225)	(0.602–1.176)	(0.410–2.971)	**(0.102–0.600)**	**(1.137–3.146)**	(0.078–5.610)	(0.564–6.349)
**Gender**							
Female	**0.729**	**0.738**	0.464	0.689	0.902	1.083	0.619
	**(0.563–0.944)**	**(0.563–0.968)**	(0.181–1.189)	(0.444–1.070)	(0.547–1.490)	(0.221–5.316)	(0.184–2.088)
**Occupation**							
Student	1.075	1.175	0.565	**4.123**	0.591	-	0.424
	(0.774–1.493)	(0.821–1.683)	(0.223–1.435)	**(1.542–11.02)**	(0.345–1.012)		(0.127–1.421)
**Nationality**							
Singaporean	0.857	0.815	1.488	0.793	0.661	0.992	1.737
	(0.666–1.102)	(0.624–1.063)	(0.619–3.578)	(0.516–1.221)	(0.397–1.102)	(0.202–4.868)	(0.515–5.856)
**Domicile**							
Hostel	1.264	**1.316**	0.583	**1.966**	1.053	1.263	0.722
	(0.981–1.629)	**(1.007–1.718)**	(0.226–1.509)	**(1.254–3.081)**	(0.639–1.736)	(0.258–6.200)	(0.214–2.435)
**Symptoms**							
Fever	**2.357**	**2.463**	0.786	**3.255**	**3.143**	1.571	1.375
	**(1.735–3.202)**	**(1.779–3.410)**	(0.333–1.854)	**(1.866–5.677)**	**(1.663–5.941)**	(0.290–8.501)	(0.408–4.637)
Chills	**1.510**	**1.504**	1.336	1.264	**2.339**	0.267	1.604
	**(1.176–1.940)**	**(1.155–1.957)**	(0.566–3.153)	(0.825–1.937)	**(1.390–3.934)**	(0.315–2.271)	(0.496–5.185)
Muscle Aches	**1.613**	**1.625**	0.845	1.531	**2.753**	0.516	1.239
	**(1.244–2.093)**	**(1.250–2.164)**	(0.356–2.003)	(0.989–2.370)	**(1.562–4.854)**	(0.095–2.793)	(0.383–4.007)
Stuffy / Runny nose	**1.327**	**1.400**	3.143	1.100	1.615	0.786	0.655
	**(1.020–1.726)**	**(1.059–1.851)**	(1.066–9.267)	(0.713–1.698)	(0.946–2.757)	(0.160–3.855)	(0.202–2.117)
Sore throat	1.264	1.253	1.615	0.897	**1.929**	1.077	1.436
	(0.956–1.671)	(0.935–1.678)	(0.597–4.370)	(0.578–1.394)	**(1.045–3.562)**	(0.199–5.821)	(0.386–5.344)
Cough	**1.425**	**1.406**	1.476	1.230	**1.722**	0.984	1.722
	**(1.102–1.843)**	**(1.073–1.842)**	(0.614–3.549)	(0.800–1.892)	**(1.023–2.899)**	(0.201–4.829)	(0.511–5.810)
Hoarse voice	1.222	1.264	1.972	0.887	**1.972**	0.263	0.751
	(0.952–1.569)	(0.972–1.645)	(0.821–4.740)	(0.573–1.372)	**(1.185–3.283)**	(0.031–2.234)	(0.223–2.534)
**Campus**							
Non-Life-science	0.861	0.858	1.004	0.807	0.818	1.931	0.901
	(0.653–1.134)	(0.642–1.147)	(0.365–2.762)	(0.506–1.288)	(0.476–1.405)	(0.228–16.37)	(0.236–3.433)

## Discussion

Influenza surveillance is important for influenza preparedness plans worldwide. Influenza-like illnesses (ILI) due to laboratory-confirmed influenza virus infections have been well studied within the temperate regions, but there are only a few studies on university students in tropical and subtropical settings. In Florida, USA, a cohort study was done on participants presenting to the university health clinic for ILIs [12]. The study was limited by small number of 60 participants with influenza infection confirmed in 63% of participants. Another study with the same number of participants was conducted in 2002 in temperate San Francisco [13] and influenza was detected in 20% of students, which is similar to our findings (32.8%). We also found a 30.6% positive rate for influenza A virus. This is similar to the positive rate for influenza A virus of 24% found in a military study in Singapore [8]. The above studies [12,13] highlight the possibility of universities acting as influenza sentinel sites, using studies similar to ours.

We found that overall, influenza does appear significantly more commonly in students living on campus within hostels, especially for the pandemic strain of subtype A/H1N1 2009. This bears out the hypothesis that influenza will be higher in close contact areas like hostels especially for novel strains of influenza. In many temperate countries, meningococcal vaccination is recommended for students living in dormitories on campus, as a preventive measure against infections that spread easily in close contact, closed communities such as hostels. Influenza is one such pathogen.

In Singapore, only high-risk groups such as the elderly, the very young (below 5), healthcare workers, or those with reduced immunity have definite recommendations for influenza vaccination. Perhaps even in tropical countries, influenza vaccination should be recommended for students who live on campus in hostels as influenza can spread even amongst healthy young adults in such close proximity.

There was no significant difference in laboratory confirmed influenza between the life sciences and the other campuses, suggesting that physical location of classes may not be an important factor for on campus transmission. This may be due to the high movement and mixing of students and staff across faculties at closed ventilation areas such as libraries, canteens, sports facilities and lecture halls.

Being a student as compared to being a staff or faculty member appeared to be a risk factor only for the pandemic strain, as proportions of infected students and non-students were not significantly different for other types and subtypes of influenza virus infections. This could be due to older staff members having some degree of immunity to the H1N1 2009 strain or perhaps to a higher degree of close contact among students compared to staff when the H1N1 2009 emerged. There were also smaller numbers of other strains in this cohort which may have led to missing an association for the other strains and subtypes.

Certain clinical symptoms were identified as being more commonly associated with laboratory confirmed influenza: fever, chills, aches and cough. These are commonly used in case definitions [14–21]. The current practice in Singapore, especially at the primary care level, is to diagnose based primarily on symptoms. Similarly, in the event of an influenza epidemic, case definitions are based primarily on symptoms. Hence, knowing whether symptoms are significant predictors of influenza positivity is of clinical significance. However, for pandemic influenza 2009, only fever was identified as being significant in distinguishing influenza from other ILI. This highlights the importance of a high index of suspicion for influenza diagnosis clinically even for those with relatively atypical presentations.

Our study had some important limitations. Data collected were from a single university, so generalization to other similar institutions would be difficult although the National University of Singapore does have a very high proportion of students from the region compared with most institutions worldwide.

The study also did not include students or staff who were clinically asymptomatic but may have been positive for influenza. Similarly, the study also did not take into account individuals who did not seek medical treatment or sought treatment outside of the UHC although anecdotally, the majority of ill staff and students do seek medical attention at the UHC.

Sampling was also affected by the university academic calendar. The majority of the samples were obtained during term periods, while few or no samples were obtained during university vacation periods. This was possibly due to a much smaller population on campus and thus smaller numbers seeking medical attention at the university health centre.

Sample sizes for type Influenza B and subtypes A/H3N2, seasonal A/H1N1 and those unable to be typed were also small, making any subgroup analyses for these subtypes and strains difficult.

## Conclusions

This study highlights the inadequacy of clinical diagnosis of influenza based on symptoms alone. In addition, we found a high concentration of laboratory confirmed influenza in students living on campus in hostels. Perhaps influenza vaccination should be recommended for students living in hostels. Given the diverse student body, the University can also act as a sentinel site for surveillance and control of influenza in large tropical institutions. This may be an important and useful strategy in containing the next pandemic.
